# A Cemented Total Hip Arthroplasty Without Osteotomy for Severe Femoral Deformity in Fibrous Dysplasia: A Case Report

**DOI:** 10.7759/cureus.103436

**Published:** 2026-02-11

**Authors:** Tetsuo Hayama, Ayano Amagami, Keigo Yonemoto, Mitsuru Saito

**Affiliations:** 1 Department of Orthopaedic Surgery, Jikei University School of Medicine, Tokyo, JPN

**Keywords:** cemented stem, fibrous dysplasia, severe femoral deformity, three-dimensional preoperative planning, total hip arthroplasty

## Abstract

A 76-year-old woman with fibrous dysplasia (FD) of the femur presented with severe deformity and secondary osteoarthritis. Total hip arthroplasty (THA) was performed using a cemented stem without femoral osteotomy, following thorough 3D preoperative planning. No intraoperative complications occurred. At the three-year follow-up, gait had significantly improved, and no loosening or fracture was observed. Cemented THA without osteotomy is a viable and less invasive option for elderly patients with FD-associated femoral deformity. This approach offers favorable implant positioning and minimizes the risk of intraoperative fracture.

## Introduction

Fibrous dysplasia (FD) is a condition first described by Lichtenstein in 1938 [1]. FD is a rare, benign bone disease in which normal bone tissue is replaced by fibroblastic and osteoblastic tissue. Lesions can occur singly or multiple times and can affect any bone in the skeleton, causing pain, deformity, and pathological fractures. Monostotic FD is more common, accounting for approximately 70% of cases, and often occurs in young people in their teens and twenties [2]. FD is caused by an activated GNAS gene mutation, which leads to constitutive activation of the Gs protein. The elevated intracellular cyclic adenosine monophosphate (cAMP) concentration in the mutant cell population leads to the overproduction of fibrous tissue in bone tissue, impairing normal bone formation [2-4]. On radiography, FD appears as a lucent lesion consisting of a mixture of fibrous tissue and woven bone, resulting in a ground-glass appearance [5]. Many lesions are clinically asymptomatic and are often discovered incidentally. Symptoms most commonly manifest as bone pain and pathological fractures. Long-term joint pathology often leads to osteoarthritis, which is caused by dysfunctional hip joint biomechanics secondary to changes in bone structure and alignment. Twelve percent of patients with FD will develop hip osteoarthritis and may require total hip arthroplasty (THA) over their lifetime [6]. These cases present challenges for orthopedic surgeons due to the severe deformity and poor bone quality associated with the disease.

We report a case of secondary osteoarthritis caused by FD with severe femoral deformity that was successfully treated with THA using a cemented stem without corrective osteotomy. Preoperative 3D planning allowed for accurate reconstruction despite the anatomical challenges. We provide a literature review of treatments for this disease and describe the management approach taken in this case.

## Case presentation

A 76-year-old woman presented with a long-standing history of right lower limb length discrepancy, which she had first noticed over 20 years earlier. At the age of 70, she underwent imaging studies for gynecological evaluation, during which FD of the right femur and secondary osteoarthritis of the right hip were identified. Over time, she developed progressive right hip pain and gait disturbance and was referred to our department for further management.

On physical examination, the right lower limb was externally rotated and noticeably shortened compared to the left (left: 79.5 cm; right: 75.0 cm). The range of motion in the right hip was limited to 70° of flexion, 5° of abduction, 0° of internal rotation, and 10° of external rotation. Single-leg stance on the affected limb was not possible. The preoperative Japanese Orthopaedic Association (JOA) hip score was 44 points.

**Figure 1:**
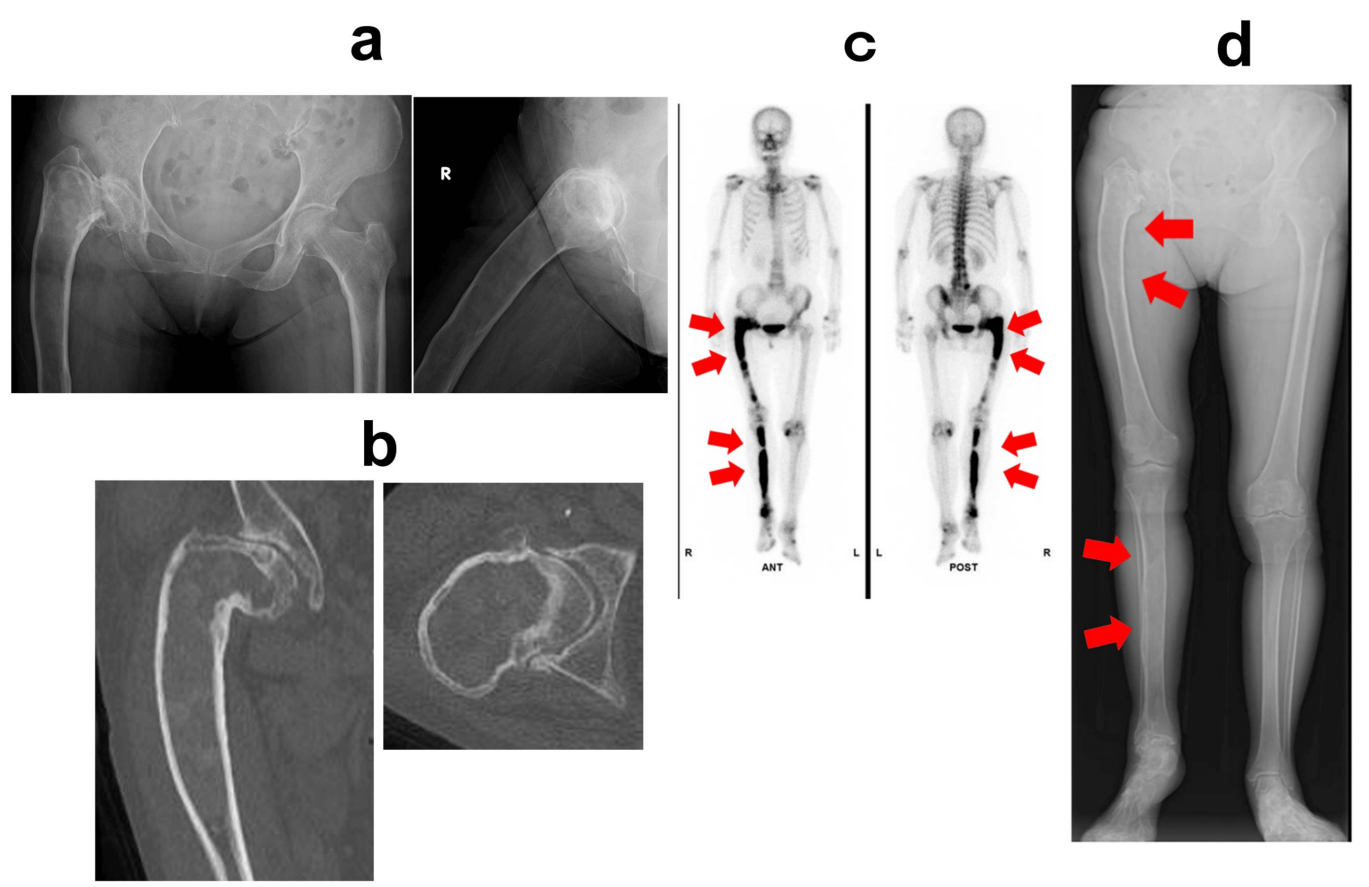
Preoperative imaging assessment (a) Plain radiograph of the hip showing severe bowing deformity of the right femur, with a ground-glass appearance of the medullary cavity and shepherd’s crook deformity with severe secondary osteoarthritis. (b) CT image showing the fibrous lesion within the right femoral medullary cavity. (c) Whole-body bone scintigraphy with technetium-99m indicates increased uptake in the right femur and tibia, consistent with polyostotic FD. (d) Full-length leg radiograph of both lower limbs showing leg length discrepancy. These findings indicate polyostotic FD and secondary osteoarthritis of the right hip. FD, fibrous dysplasia; CT, computed tomography

Plain radiographs revealed marked bowing of the right femur with a ground-glass appearance of the medullary cavity, varus deformity consistent with a “shepherd’s crook” configuration, and advanced osteoarthritic changes. Computed tomography (CT) and magnetic resonance imaging (MRI) confirmed similar findings, and a bone scintigraphy scan showed increased uptake in both the right femur and tibia (Figure 1). The diagnosis of polyostotic FD with secondary osteoarthritis was established.

**Figure 2:**
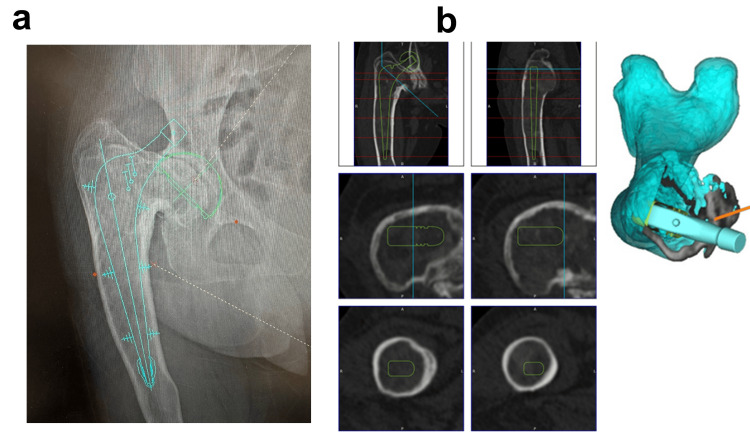
Preoperative surgical planning (a) 2D templating. (b) 3D templating using ZedHip® (LEXI Corp.). Surgical planning was based on cemented stem reconstruction without corrective osteotomy.

**Figure 3:**
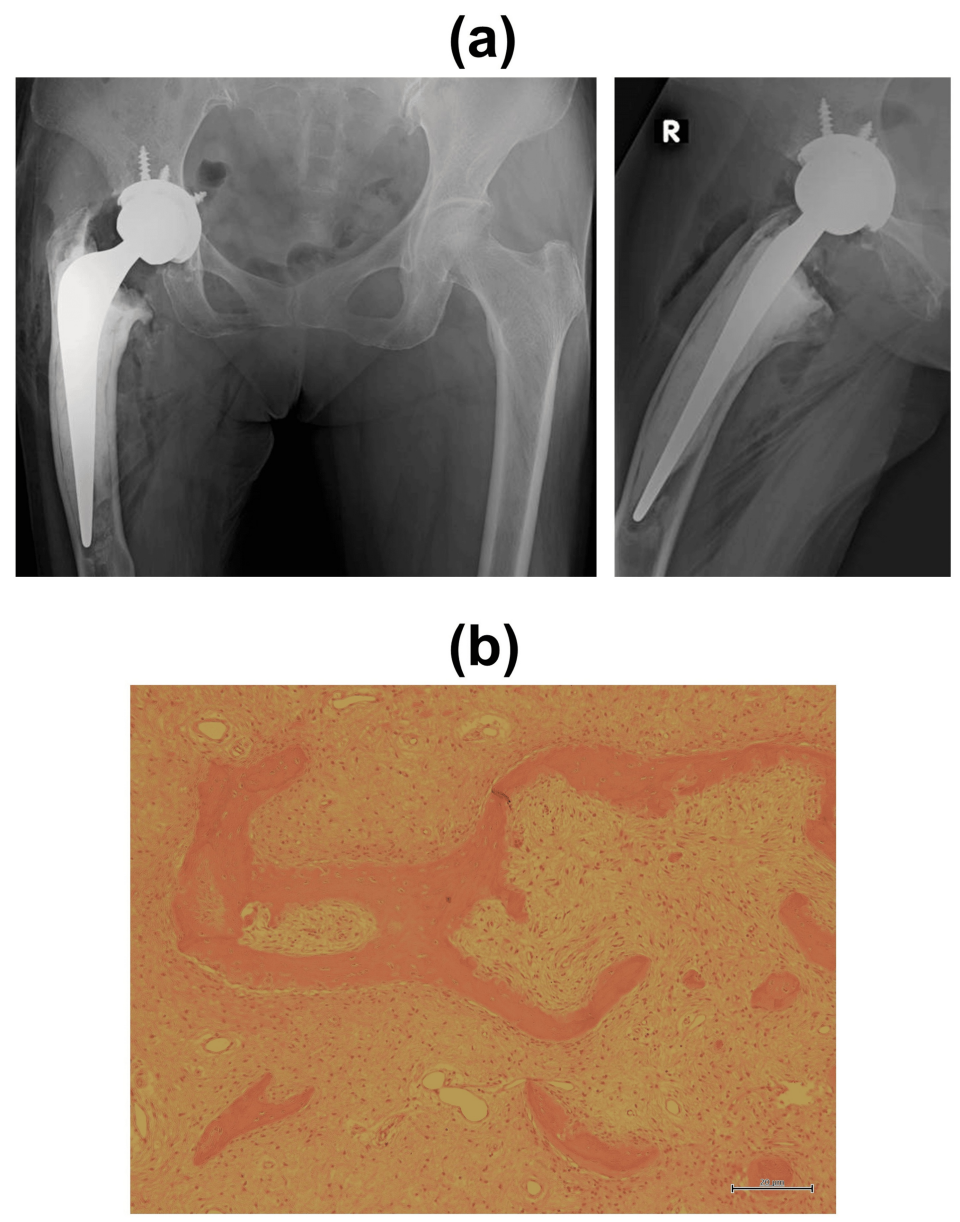
Postoperative findings (a) Postoperative radiograph of the hip showing successful stem implantation with no intraoperative complications such as fracture or excessive bleeding. (b) Histological examination (hematoxylin and eosin stain, ×40) showing irregularly arranged trabecular bone interspersed with fibrous stroma, consistent with FD. FD, fibrous dysplasia

Given the patient’s age and activity level, we selected THA using a cemented femoral stem without corrective osteotomy. A detailed preoperative plan was developed using a 3D templating system (ZedHip®) (Figure 2). The procedure was performed via a posterior approach. Intraoperatively, the medullary cavity was filled with fibrous tissue, which was thoroughly curetted. The cortical bone was contoured using a Surgairtome drill, and fluoroscopic guidance was employed to carefully prepare the femoral canal. A cemented stem was implanted according to the preoperative plan. An acetabular cementless cup (size 48 mm) with a polyethylene insert (Trident Hemispherical Acetabular Shell / X3 insert, Stryker) was inserted; the femoral component used was a size No. 3/44 offset 150 mm stem (EXETER V40, Stryker) with a 36 mm, +0 mm length Biolox delta ceramic head. The cementless cup was reinforced with three screws. The lower screw protruded slightly from the acetabulum but was covered by the iliopsoas muscle. There were no intraoperative complications such as fracture or excessive bleeding. Histopathological examination revealed irregular trabeculae within a fibrous stroma, lacking osteoblastic rimming, consistent with FD (Figure 3).

Postoperatively, standard rehabilitation protocols for THA were followed. The patient was able to ambulate with a single cane by the second postoperative week. Bone mineral density measurement by dual-energy X-ray absorptiometry (DXA) confirmed systemic osteoporosis. In addition, the intramedullary cavity of the affected femur was severely compromised by FD, leading to extremely fragile cortical bone. Furthermore, the femoral shaft exhibited a marked deformity. To reinforce bone quality and prevent postoperative periprosthetic fracture, teriparatide (a parathyroid hormone analog) was administered as a bone anabolic agent. After two years of teriparatide therapy, denosumab was initiated as a sequential antiresorptive agent. Although these agents may alleviate bone pain associated with FD, they do not reduce lesion size. In this case, the medications were primarily used to improve bone quality and prevent fragility fractures, especially around the femoral stem after THA.

**Figure 4:**
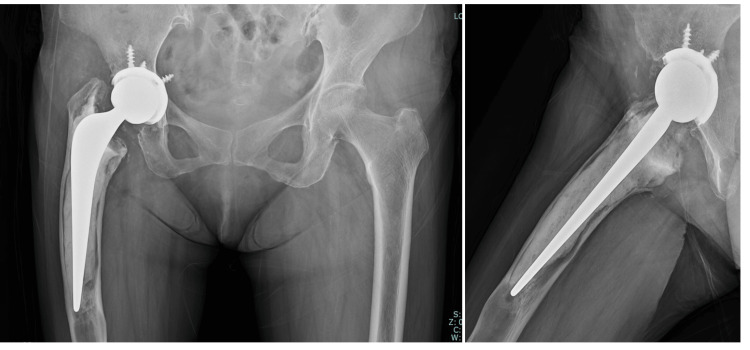
Follow-up radiograph at three years postoperatively Radiograph taken at the three-year follow-up showing stable fixation of the cemented femoral stem without signs of loosening, periprosthetic fracture, or osteolysis. The patient remained asymptomatic with independent ambulation.

At the three-year follow-up, her gait had improved significantly, the JOA score had increased to 91 points, and there was no evidence of implant loosening or periprosthetic fracture on radiographs (Figure 4).

## Discussion

FD is a rare, benign bone disorder that can severely affect the proximal femur, leading to structural deformities such as the classic “shepherd’s crook” deformity and secondary hip osteoarthritis. These cases pose a significant surgical challenge for THA, particularly in terms of deformity correction, implant fixation, and long-term stability.

There have been a few past reports on THA for FD. The papers we were able to locate are shown in Table 1 [7-11]. Sierra et al. reported that all three uncemented stems used in their cohort required early revision surgery due to loosening or poor fixation [8]. Garceau et al. noted no significant difference in outcome between cemented and uncemented reconstructions, but their cohort had a high incidence of perioperative complications, including a 90% transfusion rate and 20% rate of sciatic nerve injury [9]. On the other hand, Yao et al. reported favorable results by combining corrective osteotomy with long-stem cementless THA, supported by meticulous 3D preoperative planning [10].

**Table 1:** Summary of reported cases: THA in FD THA, total hip arthroplasty, FD, fibrous dysplasia

No.	Author (Year)	No. of Cases	Age Range	Implant Type	Key Details	Follow-Up Duration	Outcomes / Complications
1	Sierra et al., 2004 [7]	12 cases	26-57 yrs	7 Cemented / 5 cementless	-	15.7 yrs	7 hips (all cementless) required revision, monostotic was better than polyostotic disease
2	Garceau et al., 2020 [8]	10 cases	21-60 yrs	3 Cemented / 6 cementless	8 hips required allograft to the proximal femur	7 yrs	90% transfusion, 20% sciatic nerve palsy
3	Yao et al., 2019 [9]	12 cases	18-60 yrs	Cementless (long stem)	7 hips with osteotomy and bone grafting	2-6 yrs	Pain relief, independent ambulation
4	Corona et al., 2018 [10]	1 case	30 yrs	Proximal replacement cementless stem for tumors	Resection of proximal femur	4 months	Good fixation, asymptomatic
5	Moharrami et al., 2023 [11]	1 case	32 yrs	Cementless	Bilateral hips	1 year	Good fixation, asymptomatic
6	Current case	1 case	76 yrs old	Cemented	No osteotomy, 3D templating used	3 years	Improved gait, no loosening or fracture

Previous reports have demonstrated treatment outcomes in patients with FD, particularly in younger individuals aged 18 to 60 years. In contrast, this case involved a 76-year-old patient whose femur had undergone severe fragility and deformation due to long-term disease progression. Advanced age and compromised bone quality were critical considerations, leading to the selection of a cemented fixation stem. In elderly patients with FD, cemented reconstruction was considered advantageous for reducing the risk of intraoperative fracture and providing stable fixation without requiring osteotomy. With the assistance of 3D templating (ZedHip®), we were able to simulate and predict the femoral canal geometry, stem positioning, and alignment preoperatively, which contributed to a safe and accurate intraoperative stem insertion. This underscores the utility of modern 3D planning tools in complex FD-related hip reconstructions.

Cemented stems offer advantages in FD cases with compromised bone quality. They allow for easier alignment adjustment and reduce the risk of intraoperative fracture, particularly in elderly patients with fragile bone architecture. However, in this case as well, after thoroughly debriding the tumor-replaced medullary cavity, the remaining cortical bone was extremely thin and exhibited a poor, smooth surface condition. Concerns remain regarding the long-term stability of this pathological bone and cement interface, making long-term follow-up essential. Long-term concerns remain regarding the durability of the cement-fibrous bone interface and the risk of late loosening or periprosthetic fracture. Dozo et al. reported a rare case of a late periprosthetic femoral stress fracture around a cemented stem in a patient with a history of FD of the proximal femur [12]. In this case, teriparatide was initially used for the purpose of promoting bone formation and preventing fractures around the stem and was then switched to denosumab.

Consequently, continued follow-up and bone-strengthening therapies, such as the use of parathyroid hormone analogs, may play an adjunctive role in improving long-term outcomes.

## Conclusions

THA using a cemented stem without corrective osteotomy can be a safe and effective option in elderly patients with FD-associated hip arthropathy, provided that precise preoperative planning and tailored surgical execution are implemented. This case highlights the importance of a personalized surgical strategy and advanced imaging in managing technically demanding FD cases.
